# Associations Between Primary Care Providers and Staff-Reported Access Management Challenges and Patient Perceptions of Access

**DOI:** 10.1007/s11606-023-08172-w

**Published:** 2023-08-02

**Authors:** Danielle E. Rose, Lucinda B. Leung, Michael McClean, Karin M. Nelson, Idamay Curtis, Elizabeth M. Yano, Lisa V. Rubenstein, Susan E. Stockdale

**Affiliations:** 1 VA Los Angeles HSR&D Center for the Study of Healthcare Innovation, Implementation & Policy, VA Greater Los Angeles Healthcare System, Los Angeles, CA USA; 2 Geffen School of Medicine, University of California, Los Angeles (UCLA), Los Angeles, CA USA; 3 VA Puget Sound Healthcare System, Seattle, WA USA; 4 University of Washington School of Medicine, Seattle, WA USA; 5 Fielding School of Public Health, UCLA, Los Angeles, CA USA; 6 RAND Corporation, Santa Monica, CA USA; 7 Department of Psychiatry and Biobehavioral Sciences, UCLA, Los Angeles, CA USA

**Keywords:** access to care, primary care, veterans

## Abstract

**Background/Objective:**

Optimizing patients’ access to primary care is critically important but challenging. In a national survey, we asked primary care providers and staff to rate specific care processes as access management challenges and assessed whether clinics with more of these challenges had worse access outcomes.

**Methods:**

Study design: Cross sectional. National Primary Care Personnel Survey (NPCPS) (2018) participants included 6210 primary care providers (PCPs) and staff in 813 clinics (19% response rate) and 158,645 of their patients. We linked PCP and staff ratings of access management challenges to veterans’ perceived access from 2018–2019 Survey of Healthcare Experiences of Patients-Patient Centered Medical Home (SHEP-PCMH) surveys (35.6% response rate). Main measures: The NPCPS queried PCPs and staff about access management challenges. The mean overall access challenge score was 28.6, SD 6.0. The SHEP-PCMH access composite asked how often veterans reported always obtaining urgent appointments same/next day; routine appointments when desired and having medical questions answered during office hours. Analytic approach: We aggregated PCP and staff responses to clinic level, and use multi-level, multivariate logistic regressions to assess associations between clinic-level access management challenges and patient perceptions of access. We controlled for veteran-, facility-, and area-level characteristics.

**Key Results:**

Veterans at clinics with more access management challenges (> 75^th^ percentile) had a lower likelihood of reporting always receiving timely urgent care appointments (AOR: .86, 95% CI: .78–.95); always receiving routine appointments (AOR: .74, 95% CI: .67–.82); and always reporting same- or next-day answers to telephone questions (AOR: .79, 95% CI: .70–.90) compared to veterans receiving care at clinics with fewer (< 25^th^ percentile) challenges.

**Discussion/Conclusion:**

Findings show a strong relationship between higher levels of access management challenges and worse patient perceptions of access. Addressing access management challenges, particularly those associated with call center communication, may be an actionable path for improved patient experience.

## BACKGROUND

Timely access to needed care is a critical component of high-quality primary care.^1,2^ Access is foundational to building patient trust with providers, obtaining preventive care; conversely, poorer outcomes are associated with delayed or missed care.^3^ Earlier studies have focused on patient-level correlates of access^4–9^ and clinic-level interventions intended to improve patients’ access to care such as advanced access^10–12^ or patient-centered medical homes.^13–15^ Nonetheless, primary care clinic leaders face ongoing challenges to ensuring timely access to needed care given varied resources, challenges, and local contexts.^16–18^

An expert panel reached consensus on ideal access measures and access management given sparse evidence on what management interventions improve patient perceptions of access.^19–21^ The expert panel defined access management as encompassing the set of goals, evaluations, actions, and resources needed to achieve patient-centered health care services that maximize access for defined eligible populations, and optimal access management as engaging patient, providers, and teams in continuously improving care design and delivery to achieve optimal access.^21^ The expert panel also set forth a framework, based on Donabedian’s structure-process-outcome model of quality of care,^22^ to develop an evidence base of how to achieve optimal access management, given primary care practice site characteristics and supply and demand mismatches.

This study draws on the access management framework set forth by the expert panel to assess if certain processes of care impact access outcomes. We asked primary care providers and staff to rate the extent to which these processes were challenging as they attempted to ensure patients’ access to primary care during the past year. We then assessed whether these challenges were associated with patient perceptions of access (access outcomes). The access outcomes analyzed here are validated, standardized measures assessing patients’ perception of access and benchmark performance among private health insurance, Medicare, and Medicaid nationally.^23^ By assessing relationships between access management challenges and access outcomes, this study builds on previous work that explores associations between clinic-level characteristics and patient outcomes^24,25,25–28^ and strives to provide healthcare leaders with evidence-based insights about access improvements.

## METHODS

We analyzed data with individual patients’ ratings of perceived access to care (level 1) linked to primary care clinics where they received care. These responses were linked to primary care provider and staff ratings of access management challenges aggregated to clinic-level measures (level 2). This work, conducted as a non-research evaluation, was undertaken as part of the United States (U.S.) Department of Veterans Affairs, Primary Care Analytics Team (PCAT).

### Patient-Level Data Sources and Measures (Level 1)

Measures of patient perceptions of access, demographics, self-reported health status, and healthcare use were derived from VA’s Survey of Healthcare Experiences of Patients-Patient Centered Medical Home (SHEP-PCMH) survey. We analyzed SHEP-PCMH data collected August 2018 to March 2019 to align with the time that the National Primary Care Personnel Survey (NPCPS) was in the field, and 6 months after, since SHEP-PCMH asks respondents to rate care received in the previous 6 months. VA administers SHEP-PCMH with a standardized, comprehensive questionnaire to obtain veterans’ evaluations of health care received from VA clinicians. SHEP-PCMH methods are described in a report.^29^ SHEP data is widely used in VA for quality improvement.^30^ SHEP-PCMH survey items are based on the industry-standard Consumer Assessment of Healthcare Providers and Systems (CAHPS) surveys, developed by the National Committee for Quality Assurance (NCQA) and the CAHPS Consortium, sponsored by the Agency for Healthcare Research and Quality (AHRQ).^23^

We selected the three questions that comprise the CAHPS access to care composite. These questions measure ease of obtaining urgent and routine appointments and having questions answered by telephone. The questions are as follows: (1) “In the last 6 months, when you contacted this provider’s office to get an appointment for care you needed right away, how often did you get an appointment as soon as you needed?” (perceived access to urgent care appointment); (2) “In the last 6 months, when you made an appointment for a check-up or routine care with this provider, how often did you get an appointment as soon as you needed?” (perceived access to routine care appointment); and (3) “In the last 6 months, when you contacted this provider’s office during regular office hours, how often did you get an answer to your medical question that same day?” (perceived response to questions during office hours). The response options were never, sometimes, usually, and always. For each question, we created a dependent variable with responses of “always” = 1 and other responses = 0.^29,31,32^ The SHEP-PCMH survey data included measures such as age, gender, race/ethnicity, and self-rated physical health status. We included a measure of patient-provider continuity, e.g., the length of the relationship with the current provider. We hypothesized that patients with longer continuity relationships would have better perceptions of access to care.^33^ All patient characteristics included in the analysis are listed in Table 1.

**Table 1:** Characteristics of Patients who Completed the Survey of Healthcare Experiences of Patients-Patient Centered Medical Home Survey and Received at least one visit from a Primary Care Clinic in the National Primary Care Personnel Survey

	Total patient sample *N* = 158,645
	Percent (*n*)
Patient demographics	
Age	
18–34 years	1% (1487)
35–54 years	7.7% (12,145)
55–64 years	15.2% (24,063)
65 and older	76.2% (120,950)
Gender	
Male	95% (150,660)
Female	5% (7985)
Race/ethnicity	
Non-Hispanic White	80.1% (127,128)
Non-Hispanic Black	9% (14,218)
Hispanic	4.2% (6725)
American Indian or Alaska Native, Asian American or Pacific Islander, other	2.0% (3244)
Missing	4.6% (7330)
Length of relationship with primary care provider (continuity provider)	
Less than 6 months	20.2% (32,032)
At least 6 months, but less than 1 year	9.6% (15,181)
At least 1 year, but less than 3 years	27.5% (43,619)
At least 3 years, but less than 5 years	18.5% (29,404)
5 years or more	21.3% (33,855)
Missing	2.9% (4554)
Self-rated physical health status	
Excellent	6.1% (9744)
Very good	22.2% (35,239)
Good	37.6% (59,567)
Fair	25.1% (39,857)
Poor	6.1% (9747)
Missing	2.8% (4491)
Dependent variables	
In the last 6 months, when you contacted this provider’s office to get an appointment for care you needed right away, how often did you get an appointment as soon as you needed?	
Never	2.0% (3,843)
Sometimes	3.4% (6460)
Usually	7.9% (15,073)
Always	16.8% (32,107)
Missing	5.5% (10,486)
Skipped (appropriately)	64.4% (123,179)
In the last 6 months, when you made an appointment for a check-up or routine care with this provider, how often did you get an appointment as soon as you needed?	
Never	1.6% (3039)
Sometimes	4.0% (7693)
Usually	16.9% (32,370)
Always	42.7% (81,698)
Missing	6.5% (12,495)
Skipped (appropriately)	28.2% (53,853)
In the last 6 months, when you contacted this provider’s office during regular office hours, how often did you get an answer to your medical question that same day?	
Never	3.0% (5805)
Sometimes	4.7% (8969)
Usually	10.4% (19,844)
Always	19.5% (30,797)
Missing	5.9% (11,315)
Skipped (appropriately)	56.5% (107,905)

### Clinic-Level Data Sources and Measures (Level 2)

The NPCPS data was collected using a web-based, cross-sectional survey fielded August–October 2018. The 2018 study has been used to study use of access tools, management of high-risk patients, and correlates of burnout among primary care providers and staff.^34–37^ The survey methods are described elsewhere.^34,37^ The final sample included all respondents that were a core part of a primary care team, including primary care providers (physicians, physician assistants, and nurse practitioners), nurse care managers (registered nurses), licensed nurses, and administrative associates (medical support assistants or clerks). We excluded responses from extended team members such as clinical pharmacists, social workers, dieticians, and behavioral health providers because they are not directly involved in every primary care patient’s care, as core primary care team members are.

The key predictor was a clinic-level measure of access management challenges, aggregated from primary care provider and staff responses to 9 survey items. These items described different care processes that posed challenges, that is, reducing time available for direct patient care, described here as access management challenges (Table 2). Some of the processes that may have imposed additional work may have resulted from poor communication with appointment call centers. For example, some primary care providers and staff reported that receiving timely receipt of patient messages from the call center was an extreme challenge, meaning patients may not receive timely responses to questions about whether an appointment was needed or whether the issue could be resolved over the phone. Another challenge resulted when primary care providers and staff reported needing to “scrub” appointments made by call center, that is, review and determine if appointments had to be rescheduled or canceled because the appointment call center scheduled a return visit too soon, scheduled appointment(s) with the wrong primary care provider, or canceled appointments if the issue in question could be handled by telephone or email. Other processes could be associated with time-consuming care coordination challenges. For example, VA primary care providers and team members may receive calls from patients that could not reach their VA specialist or VA community care provider for lab or diagnostic test results. VA primary care teams may also be responsible for coordinating tests or labs requested by or reviewing medications prescribed by VA community care providers. VA primary care teams may also be responsible for tracking down test results or medical records from VA community care providers. Other care processes that could impact workflows were unexpected demands or needs from patients.

**Table 2:** Primary Care Clinic Characteristics from 2018 National Primary Care Personnel Survey (*n* = 813)

	Mean/SD
Primary care clinic characteristics	
Access management challenges^*^	
Have any of these issues been a challenge to your, or the PACT teamlet you spend the most time working on, efforts to improve veterans’ access to care during the past year?^†^	
Timely receipt of patient messages from the call center (phone, email, instant messenger)	2.43/.88
Scrubbing appointments made by the call center (e.g., return visit too soon, appointment with wrong PCP, primary care team member(s) could resolve issue by phone)	3.24/.96
Fielding calls or requests from patients who cannot reach their VA specialists	3.50/.83
Fielding calls or requests from patients who cannot reach their community care providers	3.23/.93
Fielding calls or requests from patients attempting to schedule routine tests (e.g., imaging) with community care providers in a clinically appropriate time frame	3.28/.96
Managing care of patients who obtained new prescriptions from community care providers to be filled by the VA pharmacy	3.45/.87
Managing care of patients who reduced or eliminated opioid use but obtained new opioid prescriptions from community care providers	3.18/.93
Obtaining outside tests and medical records performed by VA community care providers	3.54/.82
Attending to any unanticipated need for either your assigned or unassigned patients	3.27/.84
Total access management challenge score	28.59/5.99
Other characteristics	
Percent panel fullness^‡^	.92/.27
Staff: primary care clinician ratio (3:1 is considered ideal)	2.63/.72
Workload (number of primary care visits in fiscal year 2018)	26,785.65/27,850.57
Clinic Nosos risk score^§^	.94/.32

^*^Access management challenges are responses from primary care clinicians and staff summarized to the clinic level

^†^Response options: not at all challenging = 1, slightly challenging = 2, somewhat challenging = 3, moderately challenging = 4, extremely challenging = 5

^‡^The percent panel fullness is 100% or 1 if the number of patients assigned to a primary care provider is at the recommended number for each primary care provider (1200 for medical doctors or doctors of osteopathy, 900 for nurse practitioners or physician assistants). If the panel fullness measure exceeds one, the panels are considered “over”-paneled; if the panel fullness measure is less than one, the panels are considered “under”-paneled

^§^Nosos risk scores are centered around 1, meaning a veteran was expected to have costs that are the national average for VA patients. A patient with a risk score of 2.5 has an expected cost 2.5 times higher than that of the average VA patient

We included clinic-level measures from the VA Corporate Data Warehouse. These clinic-level characteristics included measures of primary care clinic staffing, workload, and risk adjustment score or case mix. We measured staffing as the number of registered nurses (care managers), licensed nurses, and medical support assistants or clerks assigned to each primary care clinician (expected ratio was 3 staff per clinician). We measured clinic-level workload as percent of panel fullness, the percent of patients assigned to each primary care clinician and team members. In VA, the panel size is typically 1200 for medical doctors and 900 for nurse practitioners or physician assistants. Since panel sizes vary, we used percent of panel fullness to have a comparable measure across primary care providers. If the panel fullness measure exceeds one, the panels are considered “over”-paneled, that is, the workload is greater than expected; if less than one, the panels are considered “under”-paneled and the workload is less than expected. We hypothesized that patients seeking care at “over”-paneled clinics would have a worse perception of access. Another measure of clinic-level workload was number of primary care visits associated with each primary care clinic in fiscal year 2018, more visits implying greater workload and worse perceived access. A third clinic-level measure was patient case mix. We used fiscal year 2018 clinic-level Nosos risk scores. Nosos risk scores are centered around 1, meaning a veteran was expected to have costs equivalent to the national average for VA patients.^38^ If the clinic-level Nosos risk score exceeded 1, the patients were assumed to be more costly and complex. We hypothesized that patients at clinics with higher Nosos risk scores would have worse perception of access.

### Statistical Analyses

The study design was cross sectional. We created clinic-level measures of access management challenges by aggregating primary care provider and staff responses. We performed bivariate analyses assessing the relationship between each access management challenge and patient perceptions of access to care. Since all access management challenges were associated with patient perceptions of access, we summarized the access management challenges into a single score. We used Cronbach’s analysis to assess internal consistency.^39^ We then performed multi-level multivariate logistic regressions, assessing associations between access management challenge scores and patient perceptions of access two ways. First, we analyzed differences for low (less than 25^th^ percentile), medium (25^th^–74^th^ percentile), or high (greater than 75^th^ percentile) access management challenge scores. To prioritize which groups of access management challenges should be targeted for intervention, we performed exploratory factor analysis with iterated principal axes, then with varimax rotations, to ascertain if the 9 challenges could be reduced into meaningful groups.^40^ We identified three factors with two access management challenges each. In multi-level multivariate models, we assessed associations of the 3 factors with each access outcome measure. For all analyses, we used Stata Version 15.^41^

## RESULTS

The overall response rate for NPCPS was 19%: 14.3% for primary care providers, 22.7% for registered nurses, 19.5% for licensed nurses, and 13.8% for clerks or medical support assistants. The final clinic-level data included 4992 responses from primary care providers and staff; their responses were aggregated to 813 clinics. Response rates for the SHEP-PCMH survey were 35.6% from August 2018 to March 2019. The aggregated clinic-level measure was linked to responses from 158,645 patients who obtained at least one visit from the 813 primary care clinics during the study period. The mean and standard deviation for each clinic-level access management challenge are presented in Table 2. Cronbach’s alpha on the items summed as a single score was 0.8757. The clinic-level mean on the access management challenge score was 28.6, SD: 6.0. Exploratory factor analysis identified 3 factors. Factor 1 was comprised of 2 items reported on a 5-point Likert scale that explained 30% of the variance, with factor loadings of 0.77 and 0.72. Factor 2 was comprised of 2 items that explained 27% of the variance, with factor loadings of 0.62 and 0.64. Factor 3 was comprised of 2 items that explained 24% of the variance, with factor loadings of 0.67 and 0.62.

For the patient perceptions of access (access outcomes), 56% of patients reported always receiving same-day or next-day (urgent care) appointment when needed; 66% reported they always got the (routine care) appointment when they made an appointment for a check-up or routine care with their provider as soon as needed; and 52% reported that when they contacted their provider’s office during regular office hours, they always got an answer to their medical question that same day (answers to medical questions).

In multi-level, multivariate models assessing associations between the access management score and patient perceptions of access (Table 3), we found that patients receiving care in primary care clinics with medium and high levels of challenges were less likely to report getting urgent care when needed [adjusted odds ratio (AOR) for clinics with a medium level of access management challenges: .86, 95% CI: 0.78–0.95; AOR for clinics with high level: 0.75, 95% CI: 0.67–0.84] or always having questions answered on the same day (AOR for clinics with medium level: 0.88, 95% CI: 0.80–0.97; AOR for clinics with high level: 0.79, 95% CI: 0.70–0.90). For routine care appointments, we only found a statistically significant difference for patients receiving care in clinics with the highest levels of access management challenges compared with the lowest level (AOR: 0.74, 95% CI: 0.67–0.82). Other patient- and clinic-level results are shown in Table 3. When we modeled access management challenges as a continuous variable, the results were similar.

**Table 3:** Multi-level Multivariate Models Assessing Associations Between access Management Challenge and Patient Perceptions of Access

	Urgent care appointment always available when needed	Routine care appointment always available when needed	Always receive answer to medical question the same day
	Adjusted odds ratio (95% confidence interval) *p* value	Adjusted odds ratio (95% confidence interval) *p* value	Adjusted odds ratio (95% confidence interval) *p* value
Primary care clinic characteristics			
Access management challenges			
Less than 25^th^ percentile	Reference	Reference	Reference
25^th^–75^th^ percentile	.86 (.78–.95) .004	.93 (.85–1.02) .148	.88 (.80–.97) .012
Greater than 75^th^ percentile	.75 (.67–.84) *p* < .001	.74 (.67–.82) *p* < .001	.79 (.70–.90) *p* < .001
Percent panel fullness (e.g., whether the number of patients assigned to a primary care provider is under or exceeds the recommended number)	.83 (.58–1.19) .309	.88 (.71–1.09) .235	.85 (.67–1.08) .175
Staff: primary care clinician ratio (3:1 is considered ideal)	.96 (.89–1.04) .307	1.00 (.94–1.06) .955	.96 (.90–1.02) .210
Workload (number of primary care visits in fiscal year 2018)	.99 (.99–.99) .039	.99 (.99–.99) .010	1.00 (.99–1.02) .210
Clinic-level Nosos risk score	1.47 (1.21–1.80) *p* < .001	.97 (.83–1.15) .756	1.43 (1.20–1.71) *p* < .001
Patient characteristics			
Age			
18–34 years	Reference	Reference	Reference
35–54 years	1.42 (1.08–1.86) .012	1.50 (1.20–1.87) *p* < .001	1.38 (1.08–1.75) .009
55–64 years	1.63 (1.25–2.11) *p* < .001	1.72 (1.39–2.13) *p* < .001	1.38 (1.10–1.74) .005
65 years and older	2.00 (1.56–2.57) *p* < .001	2.20 (1.78–2.72) *p* < .001	1.65 (1.32–2.06) *p* < .001
Gender			
Female	Reference	Reference	Reference
Male	1.20 (1.04–1.38) .015	1.12 (.99–1.27) .061	1.38 (1.18–1.62) *p* < .001
Race/ethnicity			
Non-Hispanic White	Reference	Reference	Reference
Non-Hispanic Black	.90 (.81–.99) .032	.94 (.87–1.02) .142	1.17 (1.06–1.28) .001
Hispanic	.98 (.83–1.16) .847	.92 (.82–1.03) .145	1.24 (1.07–1.45) .004
American Indian or Alaska Native, Asian American or Pacific Islander, other	.81 (.60–1.09) .157	.76 (.60–.95) .015	1.09 (.87–1.36) .473
Length of relationship with primary care provider (continuity provider)	1.05 (1.02–1.08) *p* < .001	.97 (.94–.99) .024	1.08 (1.05–1.11) *p* < .001
Self-rated physical health status			
Excellent, very good, good	Reference	Reference	Reference
Fair, poor	.56 (.52–.60) *p* < .001	.56 (.53–.60) *p* < .001	.60 (.55–.64) *p* < .001

In Table 4, we report associations between 3 access management challenge factors and patient perceptions of access. Patients at clinics reporting challenges related to the call center (factor 3: timely receipt of messages, scrubbing appointments) were less likely to perceive access as optimal (AOR for urgent care: .86, 95% CI: 0.81–0.92; AOR for routine care: .86, 95% CI: 0.81–0.92; AOR for having questions answered: 0.90, 95% CI: 0.85–0.96). Patients at clinics reporting care coordination challenges (factor 1: challenges fielding calls from patients who could not reach their VA specialist(s) or their VA community care providers) were less likely to perceive access to urgent care as optimal (AOR: .93, 95% CI: 0.87–0.99). We failed to detect statistically significant differences for the factor focused on additional workload arising from care coordination challenges (factor 2: obtaining medical information from VA community care providers and unanticipated demand).

**Table 4:** Multi-level Multivariate Models Assessing Associations Between Access Management Challenge Factors and Patient Perceptions of Access

	Urgent care appointment always available when needed	Routine care appointment always available when needed	Always receive answer to medical question the same day
	Adjusted odds ratio (95% confidence interval) *p* value	Adjusted odds ratio (95% confidence interval) *p* value	Adjusted odds ratio (95% confidence interval) *p* value
Access management challenge factors			
Factor 1: fielding calls or requests from patients who cannot reach their VA specialists; fielding calls or requests from patients who cannot reach their community care providers	.93 (.87–.99) .029	.95 (.90–1.00) .066	.98 (.92–1.04) .471
Factor 2: obtaining outside tests and medical records performed by VA community care provider(s); attending to any unanticipated need for either your assigned or unassigned patients	1.03 (.95–1.11) .487	.97 (.91–1.04) .404	.97 (.91–1.04) .466
Factor 3: timely receipt of patient messages from the call center (phone, email, instant messenger); scrubbing appointments made by call center (e.g., return visit too soon, appointment with wrong PCP, primary care team member(s) could resolve issue by phone)	.86 (.81–.92) *p* < .001	.86 (.81–.92) *p* < .001	.90 (.85–.96) .001

Models control for primary care clinic characteristics: percent panel fullness, staffing, workload (number of primary care visits in fiscal year 2018), and clinic-level Nosos risk score, and patient characteristics: age, gender, race/ethnicity, length of relationship with primary care provider (continuity provider), and self-rated physical health status

## DISCUSSION

We found statistically significant relationships between an aggregated clinic-level measure of access management challenges and patient perceptions of access. In the seminal work *Crossing the Quality Chasm*, the authors identified six aims of high-quality care, one of which was timely care.^2^ In the NASEM report, “Transforming Health Care Scheduling and Access: Getting to Now,”^3^ the authors acknowledged there are major challenges associated with providing patients timely access to needed care. While the NASEM report on access and other studies have emphasized the importance of aligning supply and demand,^21,42,43^ there is also consensus that leaders need evidence to improve access to care, especially when supply and demand were misaligned. Our study found that the relationships between access management challenges and access outcomes were similar across all 3 outcomes, after controlling for patient-, clinic-, and area-level characteristics, similar to other studies assessing relationships between clinic-level characteristics and other patient-level outcomes.^14,24,25,25–28,31^

We identified two access management challenges that are candidates for future process improvement efforts. Both involve improving communication between primary care clinics with appointment call centers. This finding is similar to that of the expert panel, where patient telephone access management was identified as one of the 8 key priorities for initiating access management improvement.^21^ There may be healthcare system issues with appointment call centers, such as inadequate staffing or inconsistent communication pathways between primary care staff and the appointment call center that may negatively impact patient experiences.^44^ Staff burnout or struggles with healthcare system challenges could result in poor-quality interactions between patients and medical support assistants working in the primary care clinic or call center and negatively impact patient perceptions of access.^36,45^ While there are many opportunities for improvement, productive approaches might include those in which patients and providers and staff partner together.^46^ This might be particularly relevant for primary care staff and appointment call centers since patients could give input on the most common problems.

In this study, we assessed determinants of patient perceptions of access, using CAHPS measures, which benchmark patient experiences for patients with private insurance, Medicare, and Medicaid. The access management challenges identified here may have relevance in non-VA settings. Other healthcare systems have identified telephone consultation as important for improving access but reported challenges with patient safety and patient experience.^47–49^

## LIMITATIONS

The study design was cross sectional; we were only able to assess associations, not causality. While we attempted to control for factors that may impact patient perceptions of access, including socio-demographic factors, length of relationship with (continuity) primary care providers, and a comprehensive set of primary care clinic processes and characteristics, we recognize there may be other factors that impact patient perceptions of access. We reported a low response rate for the NPCPS. Since we only have data on role and clinic, we lack other relevant data (e.g., primary care provider and staff demographic characteristics, burnout level, VA tenure) to assess non-response bias. A study analyzed non-response bias in an earlier wave of the NPCPS^50^ and found that there were statistically significant differences between responders and non-responders. However, these differences did not impact estimates of prevalence of burnout; we see no reason to believe there would be differential impacts on ratings of access management challenges.

## CONCLUSION

Ensuring patients’ timely access to needed care has long been recognized as an important part of high-quality primary care. We have identified a set of care processes that may detract from direct patient care (access management challenges) and are linked to variations in patient perceptions of access. We identified specific challenges, e.g., communication between appointment call centers and primary care clinics, as the potential candidates to improve patient experience. Further study is needed to identify specific challenges to communication and collaboration between VA primary care clinics and appointment call centers.

## Declarations

### Disclaimer

The views expressed are those of the authors and do not represent the views of the U.S. Department of Veterans Affairs or the United States Government.
